# Exact Solution and Large-Scale Scaling Analysis of the Imaginary Creutz–Stark Ladder

**DOI:** 10.3390/e28030259

**Published:** 2026-02-27

**Authors:** Yunyao Qi, Heng Lin, Quanfeng Lu, Dan Long, Dong Ruan, Gui-Lu Long

**Affiliations:** 1 State Key Laboratory of Low-Dimensional Quantum Physics and Department of Physics, Tsinghua University, Beijing 100084, China; qiyy21@mails.tsinghua.edu.cn (Y.Q.); lin-h21@mails.tsinghua.edu.cn (H.L.); lqf23@mails.tsinghua.edu.cn (Q.L.); 2 School of Physical Science and Technology, Tiangong University, Tianjin 300387, China; longd@tiangong.edu.cn; 3 Frontier Science Center for Quantum Information, Beijing 100084, China; 4 Beijing Academy of Quantum Information Sciences, Beijing 100193, China; 5 Beijing National Research Center for Information Science and Technology, Beijing 100084, China

**Keywords:** non-Hermitian physics, Wannier–Stark localization, Creutz ladder, imaginary stark skin effect, analytical solution, finite-size scaling

## Abstract

We present an analytical solution for the complex spectrum of a Creutz ladder subject to an imaginary Stark potential. By mapping the system to a momentum-space differential equation, we derive the closed-form solution for the momentum-space wavefunctions. We identify a distinct cross-shaped spectrum consisting of discrete localized sectors and a continuous branch of asymptotically real states. Our derivation reveals that the discrete sectors arise from a global phase winding condition, whereas the asymptotically real branch emerges when the energy magnitude is smaller than the inter-cell hopping strength, a regime in which the momentum-space wavefunction develops singularities. We demonstrate that these singularities prevent standard quantization; instead, the open boundary conditions are satisfied via a size-dependent imaginary energy component that regulates the wavefunction decay. To investigate the properties of this branch in the thermodynamic limit, we perform large-scale finite-size scaling analysis up to system sizes $L∼10^{9}$. The numerical results confirm the power-law decay of the residual imaginary energy, supporting the asymptotic reality of these states. Furthermore, scaling of the inverse participation ratio and fractal dimension indicates that these states, while exhibiting size-dependent localization in finite systems, evolve into an extended phase in the thermodynamic limit. Our results establish a theoretical framework for understanding spectral transitions in systems with imaginary Stark potentials, with potential realizations in photonic frequency synthetic dimensions.

## 1. Introduction

The study of non-Hermitian physics has revolutionized our understanding of open systems [1], particularly through the exploration of parity-time (PT) symmetry [2,3,4,5,6] and exceptional points [7,8,9]. Building on these foundations, recent research has expanded into diverse frontiers, including Liouvillian dynamics in dissipative systems [10,11,12,13,14], enhanced quantum metrology [15,16,17,18,19], and novel topological phases [20,21,22,23,24]. A pivotal development is the discovery of the non-Hermitian skin effect (NHSE) [25,26], where bulk eigenstates exponentially localize at the system boundaries, leading to the breakdown of conventional bulk–boundary correspondence [27,28]. To restore a predictive framework, the non-Bloch band theory was established, replacing the standard Brillouin zone (BZ) with a generalized Brillouin zone (GBZ) [25,29,30,31]. While the GBZ successfully describes periodic non-Hermitian systems, it relies on translational invariance. Consequently, recent research has pivoted toward systems where this symmetry is broken—such as those with disorder [32,33], quasi-periodicity [34,35,36,37,38,39,40,41,42], and impurities [43,44,45,46], which necessitates new approaches beyond the standard GBZ formulation.

One deterministic mechanism to break translational symmetry and induce novel localization phenomena is the application of a linear potential, known as the Stark potential. In the Hermitian regime, a static electric field leads to Wannier–Stark localization, where the continuous energy band splits into a discrete, equally spaced ladder of localized eigenstates [47,48,49]. Recently, the interplay between linear fields and non-Hermiticity has attracted growing interest. Investigations have revealed unique dynamical behaviors in lattices with non-reciprocal hopping and real Stark fields [50], as well as the formation of tightly bound eigenstates [51] and continuum bound states [52]. Furthermore, in dissipative lattices, the interplay between position-dependent damping and hopping has been shown to engineer an imaginary Wannier–Stark ladder [53].

**Figure 1:**
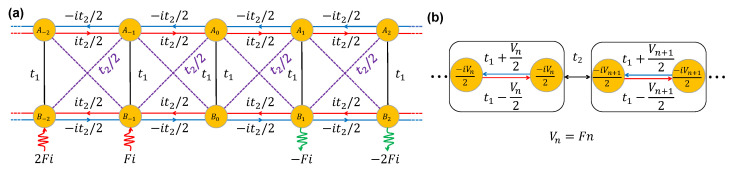
Graphical interpretation of the (**a**) original imaginary Creutz–Stark ladder and (**b**) effective nearest-neighbor hopping Hamiltonian after the local unitary rotation.

In this work, we investigate a specific non-Hermitian topology based on the Creutz ladder [54]. It is known that under specific parameter settings—vertical hopping $t_{1}$, horizontal hopping $\pm it_{2}/2$, and cross hopping $t_{2}/2$—the Creutz ladder is unitarily equivalent to the Su–Schrieffer–Heeger (SSH) model [55]. Crucially, we apply an imaginary Stark potential exclusively to one of the sublattices (the B sublattice), forming the imaginary Creutz–Stark ladder [Figure 1a]. Physically, this imaginary term serves as a phenomenological description of an open quantum or wave system subjected to a spatially graded macroscopic dissipation, such as a mode-dependent leakage rate. Historically, uniform imaginary potentials on discrete sublattices were introduced as quantum-walker models to investigate anomalous non-Hermitian dynamics like the edge burst [12]. The linearly increasing gradient version was subsequently proposed to explore boundary dynamics [56]. Specifically, for a purely lossy gradient potential (defined on sites n≥0), numerical investigations have revealed dynamical edge bursts [56], while transfer matrix methods have identified a scale-dependent localization phenomenon termed the imaginary Stark skin effect (ISSE) [57]. Theoretical foundations for solving such systems have been recently established in the Hermitian counterpart of this model [58], which utilize exact mappings to overcome constraints on mobility edges in disorder-free systems.

The sublattice-selective potential classifies this model as a “mosaic” lattice. Mosaic structures have been extensively studied for their ability to host mobility edges in Hermitian quasi-periodic systems [59,60,61]. Recently, mosaic Stark lattices [62]—featuring linear rather than quasi-periodic potentials on equally spaced sites—have been shown to generate pseudo-mobility edges in the absence of disorder or quasi-periodicity [63,64,65,66]. Subsequently, the Hermitian Creutz–Stark ladder has been proposed, demonstrating the existence of exact mobility edges by evading the Simon–Spencer theorem [58]. This rich landscape of localization phenomena has been naturally extended into the non-Hermitian domain, leading to the discovery of unique spectral features and topological phases [67,68,69].

Here, we contribute an analytical solution for the spectrum and wavefunctions of the imaginary Creutz–Stark ladder in the thermodynamic limit. Following the methodology of Ref. [58], we solve the system via a momentum-space differential equation, categorizing the spectrum into discrete localized sectors and an asymptotically real branch. A striking feature of our solution is that the momentum-space formulation directly captures the spectrum under open boundary conditions (OBCs) via the regularization of singularities, in contrast to the typical sensitivity to boundary conditions inherent to the NHSE. Consequently, our analytical results show good agreement with numerical diagonalization. Furthermore, to overcome severe finite-size effects where localized states can masquerade as extended at smaller scales [63], we perform large-scale finite-size scaling analysis up to system sizes of $L∼10^{9}$ to investigate the localization property of the asymptotically real branch. The scaling of the inverse participation ratio (IPR) and fractal dimension provides numerical support that these states—though modulated by a size-dependent localization mechanism—behave similarly to extended states in the thermodynamic limit, distinct from the localized skin modes typically observed in non-Hermitian systems under OBCs.

The remainder of this paper is organized as follows. In Section 2, we introduce the model and perform a unitary transformation to map it to an effective non-reciprocal lattice. In Section 3, we derive the analytical spectrum for the discrete energy levels. In Section 4, we analyze the asymptotically real branch and the satisfaction of boundary conditions via momentum-space singularities. Section 5 presents the large-scale scaling analysis. Finally, we conclude with a discussion of the results in Section 6.

## 2. Model and Symmetry

We consider a quasi-one-dimensional single-particle Creutz ladder [54] characterized by vertical hopping $t_{1}$, horizontal hopping $\pm it_{2}/2$, and cross hopping $t_{2}/2$. To introduce non-Hermiticity, an imaginary linear potential $V_{n}=-iFn$ is applied exclusively to the B sublattice, resulting in a configuration we term the imaginary Creutz–Stark ladder. The Hamiltonian, schematically shown in Figure 1a, is given by:

(1) $$\begin{array}{cc} H= & \sum_{n=-N}^{N}\left[t_{1}a_{n}^{†}b_{n}+\frac{t_{2}}{2}\left(a_{n}^{†}b_{n+1}+a_{n}^{†}b_{n-1}\right)+\frac{it_{2}}{2}\left(a_{n}^{†}a_{n-1}-a_{n}^{†}a_{n+1}+b_{n}^{†}b_{n+1}-b_{n}^{†}b_{n-1}\right)+H.c.\right]\\ & -\sum_{n=-N}^{N}iFnb_{n}^{†}b_{n}, \end{array}$$

where $t_{1},t_{2},F\in R^{+}$, and the lattice consists of L=2N+1 unit cells. The operators $a_{n}^{†}$ ($b_{n}^{†}$) create a particle at site *n* on the A (B) sublattice. Since the lattice indices are symmetric ($n\in \left[-N,N\right]$), reversing the sign of the imaginary potential to +iFn is equivalent to a spatial inversion n→−n, which leaves the overall spectral structure invariant.

To analyze the symmetry of this model, we first perform a local unitary rotation $U={⨁}_{n}e^{-i\pi \sigma_{x}/4}$ to transform it into a nearest-neighbor hopping model $H^{\prime }=U^{†}HU$ as shown in Figure 1b [57,58]. We first show the effective Hamiltonian respects the same symmetry as the Hermitian case [58]: $H^{\prime }$ maps to its negative under the combined transformation of chirality and parity. By applying the chiral transformation $S={⨁}_{n}\sigma_{z}$, the signs of the effective hopping terms are reversed. By applying the parity transformation P(n→−n), the effective on-site potentials undergo a sign change. Therefore, $H^{\prime }$ maps to $-H^{\prime }$ under the combined transformation SP, leading to the energy pairs (E,−E). The unique symmetry for this non-Hermitian model is the generalized PT symmetry [5]. Consider the complex conjugate operation K acting as $K^{-1}iK=-i$, which reverses the sign of the imaginary potentials. Combining with the unitary operation S which reverses the sign of the effective hopping, the effective Hamiltonian maps to its negative under the composite operation SK. Since S is unitary and K is anti-unitary, the effective Hamiltonian respects a generalized anti-PT symmetry. Hence, the energies form pairs $\left(E,-E^{*}\right)$[2,3]. Combining these two symmetries, the energies appear as $\left(E,-E,E^{*},-E^{*}\right)$. Graphically, they are distributed symmetrically in the four quadrants of the complex plane. Thus, we will focus on the energy in the first quadrant (positive real and imaginary parts) in the following context. In the following section, we will derive the spectrum analytically and demonstrate that the anti-PT symmetry is broken, i.e., the spectrum is not entirely imaginary with arbitrary field strength.

## 3. Analytical Discrete Spectrum in the Thermodynamic Limit

**Figure 2:**
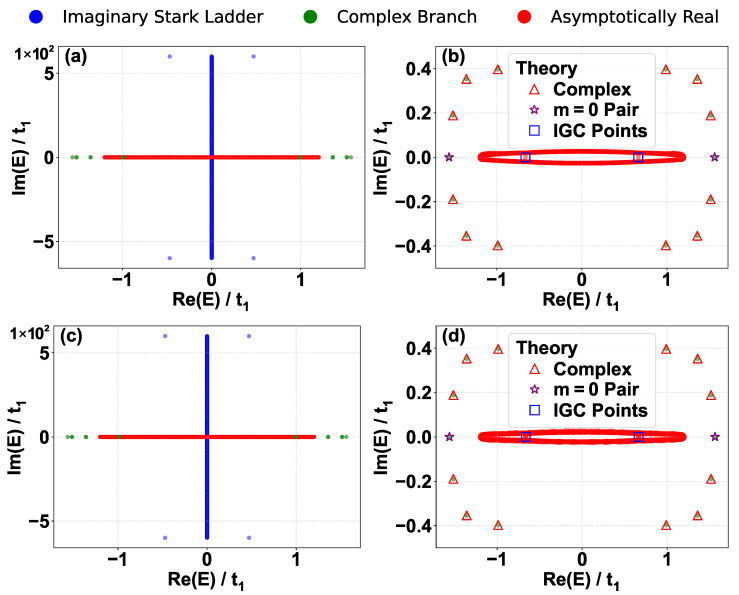
Energy spectrum of the imaginary Creutz–Stark ladder under different boundary conditions. (**a**) Global energy spectrum in the complex plane under open boundary conditions (OBCs) for F=0.6. The spectrum exhibits a characteristic cross shape, consisting of an asymptotic-real branch (red), a discrete imaginary Wannier–Stark ladder (blue), and a complex branch (green). The light blue dots at the extremities of the imaginary ladder represent modes perturbed by finite-size boundary effects. (**b**) Zoom-in of the central region of (**a**). The analytical predictions (open markers) show good agreement with numerical diagonalization (solid dots). Key features include the complex sector (red triangles), the isolated real pair at m=0 (purple stars), and the imaginary gap closing (IGC) points (blue squares) embedded within the asymptotically real branch. (**c**) Global energy spectrum under periodic boundary conditions (PBCs). (**d**) Zoom-in of the central region of (**c**). The PBC spectrum highly overlaps with the OBC spectrum, as the finite-size boundary effects (potential steps) almost identically perturb the edge-localized modes. Other parameters: L=2001 (N=1000), $t_{1}=1,\;t_{2}=1.2,\;F=0.6$.

Following the approach used in the Hermitian Creutz–Stark ladder [58], we solve for the spectrum by transforming the system into reciprocal space via the Fourier transforms $a_{n}=L^{-1/2}\sum_{k}a_{k}e^{ikn}$ and $b_{n}=L^{-1/2}\sum_{k}b_{k}e^{ikn}$. The momentum-space solution is strictly valid for periodic boundary conditions (PBCs), and in general cannot capture the spectrum under OBCs for non-Hermitian systems due to the NHSE. However, we focus on the discrete energy levels (analogous to the Wannier-Stark ladders) quantized by the single-valuedness condition in the BZ in this section. The reason that they are not sensitive to the boundary conditions is that the smooth wavefunction without singularities in the momentum space results in a localized (normalized) wavefunction in real-space [70] and hence the population at the boundaries converges to zero in the thermodynamic limit. Numerically, we find that the analytical spectrum shows good agreement with numerical diagonalization under OBCs [Figure 2a,b]. Furthermore, we have computed the numerical spectrum under PBCs [Figure 2c,d], which exhibits near-perfect overlap with the OBC spectrum. Notably, the minor discrepancies—the light blue dots at the extremities of the imaginary ladder in Figure 2a,c—are present in both cases. These represent modes localized near the edges of the lattice that are perturbed by finite-size effects. The exact momentum-space mapping implicitly assumes an infinite lattice with a continuous linear potential. Under OBCs, the lattice is abruptly truncated; under PBCs, connecting site n=N to n=−N creates a massive potential step of ΔV=2iFN. As thoroughly characterized in the standard imaginary Wannier–Stark ladder [53], states localized near these boundaries “feel” the defect unless the field *F* is large enough to shrink their localization length below a single lattice site. Consequently, these specific boundary modes deviate from the exact analytical ladder. Because the bulk states are robust and indistinguishable between the two boundary conditions, we will utilize the numerical results calculated under OBCs for subsequent comparisons in this section.

The Hamiltonian in *k*-space reads

(2) $$\begin{array}{cc} H= & \sum_{k=-\pi }^{\pi }\left[t_{1}a_{k}^{†}a_{k}+t_{2}\cos k\left(a_{k}^{†}b_{k}+b_{k}^{†}a_{k}\right)+t_{2}\sin k\left(a_{k}^{†}a_{k}-b_{k}^{†}b_{k}\right)\right]\\ & -iF\sum_{k,k^{\prime }=-\pi }^{\pi }\sum_{n=-N}^{N}ne^{i(k^{\prime }-k)n}b_{k^{\prime }}^{†}b_{k}. \end{array}$$

To handle the position-dependent term in the thermodynamic limit (L→∞), we utilize the derivative property of the delta function. The summation over *n* can be expressed as

(3) $$\begin{array}{cc} \sum_{n=-N}^{N}ne^{i(k^{\prime }-k)n} & =-i{\left.\frac{\partial }{\partial \kappa }\left(\sum_{n=-N}^{N}e^{i\kappa n}\right)\right|}_{\kappa =k^{\prime }-k}\\ & =-iL{\left.\frac{d}{d\kappa }\delta \left(\kappa \right)\right|}_{\kappa =k^{\prime }-k}\\ & =iL\delta \left(k^{\prime }-k\right)\frac{\partial }{\partial k^{\prime }}. \end{array}$$

Substituting this result into Equation (2) yields the momentum-space operator form of the imaginary potential

(4) $$-iF\sum_{k,k^{\prime }=-\pi }^{\pi }\sum_{n=-N}^{N}ne^{i(k^{\prime }-k)n}b_{k^{\prime }}^{†}b_{k}=F\sum_{k=-\pi }^{\pi }b_{k}^{†}b_{k}\frac{\partial }{\partial k}.$$

Thus, the imaginary Stark term transforms as $n\to i\partial_{k}$, preserving the block diagonal form of the Hamiltonian in reciprocal space despite the lack of translational invariance [71]. This leads to a coupled system of first-order differential equations for the sublattice wavefunctions $\phi_{A}\left(k\right)$ and $\phi_{B}\left(k\right)$:

(5) $$\left\{\begin{array}{cc} E\phi_{A}\left(k\right) & =t_{2}\sin k\;\phi_{A}\left(k\right)+\left(t_{1}+t_{2}\cos k\right)\phi_{B}\left(k\right),\\ E\phi_{B}\left(k\right) & =\left(t_{1}+t_{2}\cos k\right)\phi_{A}\left(k\right)-\left(t_{2}\sin k-F\partial_{k}\right)\phi_{B}\left(k\right). \end{array}\right.$$

By decoupling these equations, we obtain a single ordinary differential equation governing the B-sublattice component

(6) $$\begin{array}{cc} \frac{d\phi_{B}}{dk} & =V\left(k,E\right)\phi_{B}\left(k\right),\;\mathrm{with}\\ V(k,E) & =\frac{E^{2}-t_{1}^{2}-t_{2}^{2}-2t_{1}t_{2}\cos k}{F(E-t_{2}\sin k)}. \end{array}$$

We first consider the regime where no singularities exist in the integration path, i.e., $|E-t_{2}\sin k|>0$ for all k∈[−π,π]. This condition is satisfied when $|E|>t_{2}$ or *E* has a non-zero imaginary part. The no-pole condition guarantees smooth wavefunction in the momentum space and discrete energy levels, which is the focus of this section. We will discuss the case with singularities in the next section. We decompose the integral of the potential V(k,E) into two parts:

(7) $$\int V\left(k,E\right)dk=\int \frac{E^{2}-t_{1}^{2}-t_{2}^{2}}{F(E-t_{2}\sin q)}\;dq-\int \frac{2t_{1}t_{2}\cos q}{F(E-t_{2}\sin q)}dq.$$

The second term is the integral of an exact differential, yielding $\left(2t_{1}/F\right)\ln\left(\alpha -\sin k\right)$, where $\alpha =E/t_{2}$. The first term is evaluated using the standard Weierstrass substitution x=tan(q/2), which transforms the differential as $dq=\frac{2dx}{1+x^{2}}$ and $\sin q=\frac{2x}{1+x^{2}}$:

(8) $$\begin{array}{cc} \int \frac{dq}{\alpha -\sin q} & =\int \frac{2dx}{\alpha (1+x^{2})-2x}\\ & =\frac{1}{\sqrt{1-\alpha^{2}}}\ln\left(\frac{\alpha \tan\left(k/2\right)-1-\sqrt{1-\alpha^{2}}}{\alpha \tan\left(k/2\right)-1+\sqrt{1-\alpha^{2}}}\right). \end{array}$$

Combining these results gives the closed-form wavefunction:

(9) $$\phi_{B}\left(k\right)=C{\left(\frac{\alpha \tan\frac{k}{2}-1-\sqrt{1-\alpha^{2}}}{\alpha \tan\frac{k}{2}-1+\sqrt{1-\alpha^{2}}}\right)}^{\frac{E^{2}-t_{1}^{2}-t_{2}^{2}}{Ft_{2}\sqrt{1-\alpha^{2}}}}{\left(\alpha -\sin k\right)}^{\frac{2t_{1}}{F}},$$

where C is the integration constant. The physical validity of the wavefunction requires single-valuedness in the BZ, i.e., $\phi_{B}\left(k+2\pi \right)=\phi_{B}\left(k\right)$.

This condition imposes a quantization constraint on the phase accumulation. For real energies satisfying |α|>1, the argument of the logarithm accumulates a phase of 2π across the BZ. Thus, the prefactor in the exponent must be an integer:

(10) $$\frac{E^{2}-t_{1}^{2}-t_{2}^{2}}{Ft_{2}\sqrt{1-\alpha^{2}}}=m,\;m\in Z.$$

For complex energies, we verify this condition via the residue theorem. Defining $\beta =e^{iq}$, the loop integral of the first term in Equation (7) becomes

(11) $$I=\oint_{|\beta |=1}\frac{E^{2}-t_{1}^{2}-t_{2}^{2}}{i\beta F(E+it_{2}\left(\beta -\beta^{-1}\right)/2)}\;d\beta =\oint_{|\beta |=1}\frac{2(E^{2}-t_{1}^{2}-t_{2}^{2})}{-t_{2}F\left(\beta^{2}-2iE\beta /t_{2}-1\right)}\;d\beta .$$

The roots of the denominator $\beta^{2}-2iE\beta /t_{2}-1=0$ are

(12) $$\beta_{\pm }=i\alpha \pm \sqrt{1-\alpha^{2}},$$

which satisfy $\beta_{+}\beta_{-}=-1$. Consequently, one root ($\beta_{-}$) lies inside the unit circle while the other ($\beta_{+}$) lies outside, provided no poles lie exactly on the circle. By the residue theorem:

(13) $$I=2\pi i\cdot \mathrm{Res}\left(\beta_{-}\right)=2\pi i\left(\frac{2(E^{2}-t_{1}^{2}-t_{2}^{2})}{-t_{2}F\left(\beta_{-}-\beta_{+}\right)}\right)=\frac{2\pi i(E^{2}-t_{1}^{2}-t_{2}^{2})}{Ft_{2}\sqrt{1-\alpha^{2}}}.$$

The single-valuedness condition requires this phase to be a multiple of 2πi, which recovers Equation (10). Solving for *E*, we obtain the explicit discrete spectrum

(14) $$E_{m}^{2}=t_{1}^{2}+t_{2}^{2}-\frac{mF}{2}\left(mF+\sqrt{m^{2}F^{2}-4t_{1}^{2}}\right),\;m\in Z.$$

While Equation (14) formally allows a ± sign for the square root, we fix the positive sign and allow the integer *m* to take both positive and negative values to cover all spectral branches. We classify the spectrum into three sectors based on quantum number *m*.

### 3.1. Sector I: The Imaginary Wannier–Stark Ladder (m≫1)

**Figure 3:**
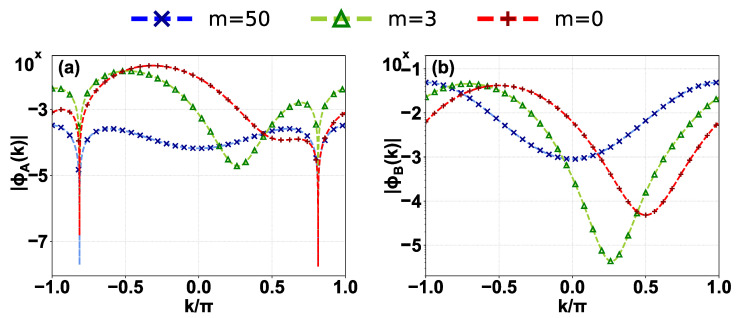
Amplitude of the momentum-space wavefunctions $|\phi_{A,B}\left(k\right)|$ on a logarithmic scale for representative discrete states: (**a**) A sublattice and (**b**) B sublattice. Comparison between numerical diagonalization (markers) and analytical derivation (dashed lines) for the real pair (m=0, red +), the complex branch (m=3, green ∆), and the imaginary Wannier–Stark ladder (m=50, blue ×). The analytical curves are derived from Equation (9).

For large integers *m*, the term under the square root dominates, and $E_{m}^{2}\approx -m^{2}F^{2}$. This yields the asymptotic behavior $E_{m}\approx \pm imF$, representing an infinite ladder of purely imaginary eigenvalues. These states correspond to modes deeply localized by the imaginary potential gradient on the B sublattice, forming the vertical linear feature in Figure 2a. We numerically confirm the validity of Equation (9) for these discrete states by comparing the theoretical amplitude of the momentum-space wavefunction with numerical result of a representative state with m=50 in Figure 3 (blue lines and crosses). For the imaginary ladder state (m=50), the wavefunction is smooth and periodic in the BZ, consistent with its strong localization in real space.

### 3.2. Sector II: The Complex Branch (Small |m|)

For indices satisfying $|m|F<2t_{1}$, the term inside the square root in Equation (14) is negative. This generates a finite set of complex conjugate pairs. In the complex plane, these eigenvalues are distributed around the real axis [red triangles in Figure 2b]. The corresponding wavefunction is smooth and agrees well with the numerical result on a representative with m=3 in Figure 3 (green lines and triangles).

### 3.3. Sector III: Real Pair (m=0)

At m=0, Equation (14) yields exactly two real eigenvalues

(15) $$E_{0}^{\pm }=\pm \sqrt{t_{1}^{2}+t_{2}^{2}}.$$

These values are independent of *F*. The analytical prediction from Equation (15) (purple stars) agrees well with the numerical results in Figure 2b, and the corresponding momentum-space wavefunctions derived from Equation (9) agree well with the numerical results in Figure 3 (red lines and plus signs). We now prove that these are the only discrete real energies satisfying the no-pole condition $|E|>t_{2}$.

Real solutions for m≠0 require the discriminant $m^{2}F^{2}-4t_{1}^{2}\geq 0$. We analyze the monotonicity of $E_{m}^{2}$ with respect to *m*. Differentiating Equation (14):

(16) $$\frac{\partial E_{m}^{2}}{\partial m}=-mF^{2}\left(1+\frac{m^{2}F^{2}-2t_{1}^{2}}{mF\sqrt{m^{2}F^{2}-4t_{1}^{2}}}\right).$$

For positive $m\geq 2t_{1}/F$, clearly $\partial E_{m}^{2}/\partial m<0$. The maximum value occurs at the boundary $m=2t_{1}/F$, where $E^{2}=t_{2}^{2}-t_{1}^{2}<t_{2}^{2}$. Thus, no real solutions with $|E|>t_{2}$ exist for m>0.

For negative $m\leq -2t_{1}/F$, we note that $\left(m^{2}F^{2}-2t_{1}^{2}\right)>\left|m\right|F\sqrt{m^{2}F^{2}-4t_{1}^{2}}$. Consequently, the term in the parentheses is negative, and combined with the negative prefactor $-mF^{2}$, we have $\partial E_{m}^{2}/\partial m<0$. The value of $E_{m}^{2}$ increases as m→−∞. A Taylor expansion gives the asymptotic limit

(17) $$E_{m}^{2}≃t_{1}^{2}+t_{2}^{2}-\frac{mF}{2}\left[mF+|m|F\left(1-\frac{2t_{1}^{2}}{m^{2}F^{2}}-\frac{2t_{1}^{4}}{m^{4}F^{4}}\right)\right]\to t_{2}^{2}-\frac{t_{1}^{4}}{m^{2}F^{2}}.$$

Since the asymptotic limit approaches $t_{2}^{2}$ from below, all real solutions for m<0 satisfy $E_{m}^{2}<t_{2}^{2}$. Therefore, they violate the no-pole condition $|E|>t_{2}$ required for the validity of the discrete solution. This proves that $E_{0}^{\pm }$ are the unique discrete real states in the system. The existence of m=0 real energy pairs for arbitrary *F* leads to the breaking of generalized anti-PT symmetry since the spectrum is not entirely imaginary.

Notably, a general feature of all discrete states shown in Figure 3a is the presence of nodes in $|\phi_{A}\left(k\right)|$, while $|\phi_{B}\left(k\right)|$ remains node-less. This structural behavior originates directly from the coupled equations in Equation (5). Since the no-pole condition requires $E-t_{2}\sin k\neq 0$ for all discrete levels, the relation $\phi_{A}\left(k\right)=\frac{t_{1}+t_{2}\cos k}{E-t_{2}\sin k}\phi_{B}\left(k\right)$ forces $\phi_{A}\left(k\right)$ to vanish exactly at the zeros of $\left(t_{1}+t_{2}\cos k\right)$. Conversely, the analytical solution for $\phi_{B}\left(k\right)$ in Equation (9) remains strictly non-zero for real *k* under the no-pole condition, leaving it node-less.

## 4. The Asymptotically Real Branch

In contrast to the discrete sectors derived in Section 3, the spectrum exhibits a continuous branch for asymptotically real energies satisfying $|E|<t_{2}$. In this regime, the potential V(k,E) in Equation (6) develops singularities at resonant momenta $k_{1},k_{2}$ where $E=t_{2}\sin k_{1,2}$, originating from a resonance with the dispersion band of the isolated A sublattice. Physically, this means that the energy of the system matches the Hermitian band $E\left(k\right)=t_{2}\sin k$ of the A chain, causing the population to be overwhelmingly dominated by the Bloch wave on the A sublattice. These singularities reside on the real axis of the complex *k*-plane (or the unit circle in the β-plane), preventing the formation of the global phase winding required for the quantization condition in Equation (10) since traversing the BZ is impossible due to the existence of the singularities. Instead, the two poles form the new boundaries and the analytical solution Equation (9) is valid on each interval separated by the poles. This enables the existence of states with arbitrary energy satisfying E∈R and $|E|<t_{2}$, forming a dense continuum on the real axis [horizontal red line in Figure 2a]. The name asymptotically real branch originates from the numerical result that energies in this branch possess small but nonzero imaginary parts [red dots close to the real axis in the zoomed-in plot Figure 2b]. The imaginary part is size-dependent and decays with system size. In this section, we derive the properties of these states and explain how they satisfy the OBC through a size-dependent imaginary energy component.

### 4.1. Singularities in Momentum Space

Near a resonant pole $k_{i}$, the potential behaves as:

(18) $$V\left(k,E\right)≃\frac{{\left(t_{1}+t_{2}\cos k_{i}\right)}^{2}}{Ft_{2}\cos k_{i}\left(k-k_{i}\right)}\;:=\;\xi_{i}{\left(k-k_{i}\right)}^{-1},\;i=1,2.$$

Integrating this potential yields a logarithmic term, which upon exponentiation leads to an algebraic divergence in the wavefunction: $\phi_{B}\left(k\right)∼{\left(k-k_{i}\right)}^{\xi_{i}}$. Since $\sin k_{1}=\sin k_{2}$ and $\cos k_{1}=-\cos k_{2}$, the exponents satisfy $\xi_{1}\xi_{2}=-{\left(t_{1}^{2}-t_{2}^{2}\cos^{2}k_{1}\right)}^{2}/\left(F^{2}t_{2}^{2}\cos^{2}k_{1}\right)<0$. This implies that the wavefunction $\phi_{B}\left(k\right)$ converges at one pole (let us denote it as $k_{1}$) and diverges at the other ($k_{2}$). Through the relation $\phi_{A}\left(k\right)=\left(t_{1}+t_{2}\cos k\right){\left(E-t_{2}\sin k\right)}^{-1}\phi_{B}\left(k\right)$, the A-sublattice wavefunction scales as ${\left(k-k_{i}\right)}^{\xi_{i}-1}$, ensuring divergence at $k_{2}$ for both sublattices.

A special case arises at the imaginary gap closing (IGC) points [72], $E_{\mathrm{IGC}}^{\pm }=\pm \sqrt{t_{2}^{2}-t_{1}^{2}}$ (for $t_{2}>t_{1}$). Here, the Stark effect is nullified because $t_{1}+t_{2}\cos k_{2}=0$ and $E=t_{2}\sin k_{2}$ are simultaneously satisfied, causing the wavefunction amplitude to vanish on the B sublattice. This results in robust extended states and exact real energies [Squares in Figure 2b].

**Figure 4:**
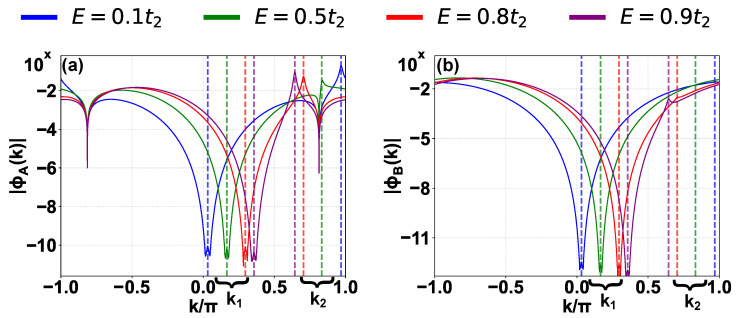
Numerical results of momentum-space wavefunctions for the asymptotically real branch ($|E|<t_{2}$) for (**a**) A sublattice and (**b**) B sublattice at four selected energies $E/t_{2}\in \left\{0.1,0.5,0.8,0.9\right\}$. Vertical dashed lines indicate the theoretical resonant momenta $k_{1,2}$ satisfying $E=t_{2}\sin k_{1,2}$. For the selected positive energies, the resonant momenta less than π/2 correspond to the converging points ($k_{1}$) where the wavefunctions converge to 0; the resonant momenta greater than π/2 correspond to the diverging points ($k_{2}$) where the wavefunctions diverge.

The divergences in momentum space are the signature of these states. We verify this in Figure 4 for the four representative states sampled from the asymptotically real branch. While all four representative states exhibit convergent behavior at the predicted points $k_{1}$ (marked with vertical dashed lines less than π/2), the divergence behavior at predicted points $k_{2}$ (marked with vertical dashed lines greater than π/2) is generally much weaker and effectively vanishes for the state with Re(E)≈0.5t2 (green solid line). This asymmetry can be well understood from the detailed analysis for the two poles. For the chosen parameters $t_{1},t_{2},F\in R^{+}$ and Re(E)>0, we have $k_{1}<\pi /2<k_{2}$ from $E=t_{2}\sin k_{1,2}$, and $\xi_{1}>\left|\xi_{2}\right|$ from the definition Equation (18). Thus, the rate of convergence at $k_{1}$ significantly exceeds the rate of divergence at $k_{2}$. Notably, for Re(E)≈0.5t2, which lies close to the IGC point $E=\sqrt{t_{2}^{2}-t_{1}^{2}}\approx 0.66t_{2}$, $\xi_{2}\approx -0.013$ has the smallest absolute value among the four states, resulting in the suppression of the divergence in finite-size systems.

### 4.2. Open Boundary Conditions and Real-Space Decay

The previous argument that a smooth momentum-space wavefunction leads to localized states in real-space and insensitivity to the boundary conditions is no longer valid for the asymptotically real branch due to the existence of singularities. Here we analyze the real-space behavior of this branch under OBCs. First, we provide a qualitative explanation that there is no traditional NHSE in this branch. The NHSE typically arises when the PBC spectrum forms a loop with a non-zero winding number encircling the OBC spectrum [73,74,75,76]. Here, the PBC spectrum is real in the thermodynamic limit; since a line on the real axis encloses no area, the spectral winding number vanishes.

However, the states must still satisfy the OBCs (ψ→0 at boundaries). We now analyze how the residual imaginary energy component in finite systems enables this. Since the branch is dominated by the A sublattice, we examine $\psi_{A}\left(n\right)$. According to the properties of Fourier transforms for generalized functions [77], the asymptotic behavior of $\psi_{A}\left(n\right)$ as |n|→∞ is dominated by the inverse Fourier transform of the singularities ${\left(k-k_{i}\right)}^{\xi_{i}-1}$ (i=1,2). This transform is determined by the regularization of the poles in the complex plane [78]

(19) $$F^{-1}\left[{\left(k-k_{i}\pm i0\right)}^{\xi_{i}-1}\right]\propto \Theta \left(\pm n\right){\left|n\right|}^{-\xi_{i}}e^{ik_{i}n},$$

with F denoting the Fourier transform, i0 denoting an infinitesimal shift in the complex plane and Θ(x) being the Heaviside step function. Physically, the finite system size induces a small imaginary component to the energy, $E=E_{0}+i\delta$, which shifts the resonant momenta $k_{i}$ off the real axis, selecting the ±i0 regularization.

To be explicit, consider the roots in the β-plane ($\beta =e^{ik}$). Using Equation (12) with $E=E_{0}+i\delta$ (assuming $E_{0},\delta >0$), we obtain the following approximation for $\delta \ll t_{2}$

(20) $$\beta_{\pm }\left(E_{0},\delta \right)≃\frac{iE_{0}-\delta }{t_{2}}\pm \sqrt{1-\frac{E_{0}^{2}-\delta^{2}}{t_{2}^{2}}}\left(1-\frac{iE_{0}\delta }{t_{2}^{2}-E_{0}^{2}+\delta^{2}}\right).$$

**Figure 5:**
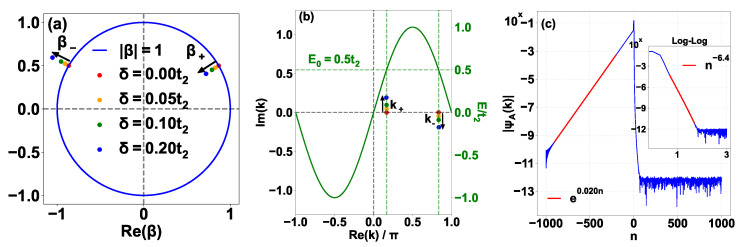
Mechanism of boundary condition satisfaction for the asymptotically real branch. (**a**) Trajectory of the roots $\beta_{\pm }\left(E\right)$ in the complex plane as the energy acquires a finite imaginary component δ (where $E=0.5t_{2}+i\delta$). The blue line represents the unit circle (|β|=1) and the colored dots represent $\beta_{\pm }$ under different values of δ. As δ increases, $\beta_{+}$ moves inside the unit circle while $\beta_{-}$ moves outside. (**b**) Corresponding trajectory of the resonant momenta $k_{\pm }=-i\ln\beta_{\pm }$ in the complex *k*-plane. The solid green curve represents the dispersion $E=t_{2}\sin k$. Its intersections with the dashed horizontal line corresponding to $E_{0}=0.5t_{2}$ (right axis) determine the purely real resonant momenta for δ=0 (indicated by the vertical green dashed lines). As the energy acquires the imaginary component δ, these resonant momenta shift off the real axis and develop non-zero imaginary parts (left axis, colored dots). The imaginary parts Im(k+) and Im(k−) develop opposite signs, ensuring wavefunction decay at opposite boundaries. (**c**) Spatial profile of the A-sublattice wavefunction amplitude $|\psi_{A}\left(n\right)|$ (blue) obtained via exact diagonalization ($L=2001,E\approx 0.5t_{2}$). Main Panel: Log-linear plot showing the exponential decay $e^{-\eta |n|}$ for n<0. The red line is an exponential fit $|\psi_{A}\left(n\right)|∼e^{0.020n}$, consistent with the theoretical prediction η=|Im(k−)|. Inset: Log–log plot of the decay for n>0, compensated by the exponential factor. The red line represents a power-law fit $|\psi_{A}\left(n\right)|e^{-0.020n}∼n^{-6.4}$, showing agreement with the analytical exponent $\xi_{1}\approx -6.7$. Parameters: $t_{1}=1,\;t_{2}=1.2,\;F=0.6$.

We observe that 0<Im[β+]<Im[β−]. Since $\beta_{+}\beta_{-}=-1$, one root ($\beta_{+}$) moves inside the unit circle while the other ($\beta_{-}$) moves outside, as shown in Figure 5a. Transforming back to momentum space via $k_{\pm }=-i\ln\beta_{\pm }$, we find Im(k−)<0 and Im(k+)>0 [Figure 5b]. Matching these to the resonant momenta (where $k_{-}$ corresponds to the diverging point $k_{2}$ and $k_{+}$ to the converging point $k_{1}$ for our parameters since $k_{1}<\pi /2<k_{2}$ for entirely real energies), Equation (19) yields the asymptotic real-space profile:

(21) $$\psi_{A}\left(n\right)≃\left\{\begin{array}{cc} An^{-\xi_{1}}e^{ik_{1}n},\; & n\gg 1,\\ {B|n|}^{-\xi_{2}}e^{ik_{2}n},\; & n\ll -1, \end{array}\right.$$

where A,B are integration constants. The opposite signs of Im(k1,2) ensure exponential decay at both boundaries (n→±∞), satisfying the OBCs. As the system size L→∞, the imaginary part δ→0, causing the localization length to diverge. Thus, the state approaches an extended distribution on the A sublattice. Another feature is that the real-space wavefunction is dominated by a single momentum component under the OBC, which is different from periodic systems where the OBC is satisfied by the linear combination of two counter-propagating waves. These two properties are consistent with the previously investigated ISSE with a purely lossy lattice, i.e., taking only the positive lattice indices, and obtaining only negative imaginary parts in the energies [57].

We numerically verify this behavior in Figure 5c for a representative state with $E\approx 0.5t_{2}$ in a system of size L=2001. The exact diagonalization yields δ=0.021. Using Equation (18), we obtain theoretical poles $k_{1,2}$ with imaginary parts ≈±0.020 (the imaginary parts of $k_{1}$ and $k_{2}$ are opposite since the corresponding $\beta_{1,2}$ satisfies $\beta_{1}\beta_{2}=-1$) and exponents $\xi_{1}\approx 6.7$, $\xi_{2}\approx -0.023$. For n<0, the wavefunction is dominated by the exponential decay e−Im(k2)n. An exponential fit yields a decay rate of 0.020, consistent with the theoretical |Im(k2)|. For n>0, the behavior is a fast power-law decay $n^{-\xi_{1}}$ modulated by a slow exponential envelope since $\xi_{1}$ is much larger than $|\xi_{2}|$. After compensating for the exponential factor eIm(k1)n, a power-law fit (inset) yields an exponent of −6.4, which is close to the theoretical prediction $-\xi_{1}\approx -6.7$. The fitting is conducted on relatively small indices ranging from n=5 to n=50 where the amplitude is not too small (otherwise the error can be significant due to precision limits), which can explain the relatively large difference between theoretical and numerical results since Equation (21) only governs the asymptotic behavior at large |n|.

## 5. Scaling Analysis

While the wavefunction analysis offers local insights, the scaling behaviors of states from different branches are also crucial to fully characterize their spatial extent in the thermodynamic limit. To quantitatively distinguish whether the states in the asymptotically real branch resemble extended, localized, or critical states in the thermodynamic limit, we utilize the inverse participation ratio (IPR), defined as [79]

(22) $$\mathrm{IPR}=\sum_{n}{\left|\psi_{n}\right|}^{4}/{\left(\sum_{n}{\left|\psi_{n}\right|}^{2}\right)}^{2},$$

and the corresponding fractal dimension [80]

(23) $$D_{2}=-\ln\left(\mathrm{IPR}\right)/\ln L.$$

Although the system does not possess a geometric fractal structure, $D_{2}$ serves as a standard and robust metric for identifying localization phases: $D_{2}\to 0$ indicates localized states, $D_{2}\to 1$ indicates extended states, and intermediate values characterize critical states [63,79]. To perform this scaling analysis, we extend our numerical simulations to system sizes up to $L\approx 10^{9}$. Handling such large-scale non-Hermitian matrices is computationally demanding; we address this by utilizing the tridiagonal structure of the effective Hamiltonian after the unitary rotation. Following the approach detailed in our previous work [58], we employ a shift-invert Arnoldi algorithm [81] combined with a disk-backed storage scheme (utilizing memory mapping techniques) to circumvent memory limitations (see Appendix A for implementation details).

**Figure 6:**
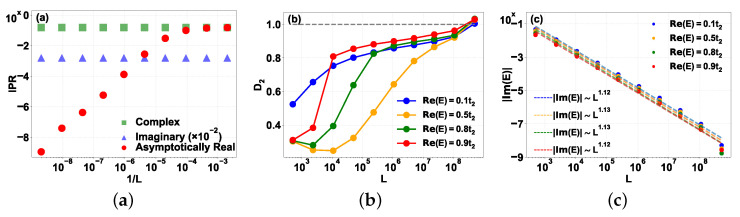
Finite-size scaling analysis of the representative states. (**a**) Inverse participation ratio (IPR) as a function of inverse system size 1/L for three representative states: the imaginary Wannier–Stark ladder (m=50, blue triangles, scaled by $10^{-2}$ for visibility), the complex branch (m=3, green squares), and the asymptotically real branch (Re(E)≈0.5t2, red circles). (**b**) Evolution of the fractal dimension $D_{2}$ with system size *L* for four representative states within the asymptotically real branch (Re(E)/t2∈{0.1,0.5,0.8,0.9}). The gray dashed line indicates the extended limit $D_{2}=1$. (**c**) Scaling of the absolute imaginary energy component |Im(E)| versus system size *L* for the same four states shown in (**b**). Dashed lines represent power-law fits |Im(E)|∝L−γ. Parameters: $t_{1}=1,\;t_{2}=1.2,\;F=0.6$, with *L* ranging from roughly $10^{2}$ to $10^{9}$.

In Figure 6a, we plot the IPR as a function of the inverse system size 1/L. The distinct behaviors of the spectral sectors are evident: states from the imaginary ladder (m=50) and the complex branch (m=3) exhibit an IPR that saturates to a finite value, characteristic of localized states. In contrast, the IPR for the asymptotically real state ($E\approx 0.5t_{2}$) remains constant for small system sizes but scales almost linearly with 1/L in the regime $L>10^{5}$, indicating that the wavefunction spreads over the entire lattice volume in the thermodynamic limit.

We further characterize the spatial extent of the wavefunctions from the asymptotically real branch using the fractal dimension $D_{2}$, shown in Figure 6b. As *L* increases, $D_{2}$ for all sampled states within the asymptotically real branch tends toward unity, supporting the conclusion that they behave as extended states in the thermodynamic limit. Notably, the state near the IGC point ($E\approx 0.5t_{2}$, yellow line) exhibits a non-monotonic crossover: at small sizes ($L<10^{4}$), $D_{2}$ remains low, reflecting the weak algebraic divergence discussed in Section 4; however, at sufficiently large scales ($L>10^{6}$), $D_{2}$ rises rapidly and converges toward the extended limit. Crucially, while our asymptotic real-space analysis predicts an exponential decay envelope, the $D_{2}\to 1$ scaling provides fundamentally new insight by confirming that the localization length diverges in the thermodynamic limit. This highlights the necessity of large-scale scaling to resolve the extended nature of the states in the asymptotically real branch, as finite-size effects can cause states to masquerade as localized or extended up to $L∼10^{5}$ [63].

Finally, we investigate the “asymptotic reality” of this branch in Figure 6c. In finite systems, these states possess a residual imaginary energy component. We track |Im(E)| for four representative states (Re(E)/t2∈{0.1,0.5,0.8,0.9}) as *L* increases. The data reveals a power-law decay with fitted exponents γ ranging from 1.12 to 1.13. While the exact theoretical value of γ in the strict L→∞ limit remains an open question, we note that the perturbative approach used to obtain the scaling of Im(E) in Ref. [57] yields a strictly zero imaginary correction for our model. This is because the symmetries of our setup enforce $\left(E,E^{*}\right)$ eigenvalue pairing; a non-degenerate real energy level cannot continuously acquire an imaginary part to any finite order of standard perturbation theory without a partner to form a complex conjugate pair. Thus, the emergence of the imaginary energy component is a non-perturbative effect driven entirely by the finite-size boundaries truncating the tails of the wavefunctions. Nevertheless, the strictly positive value of the fitted exponent confirms that these eigenvalues approach the real axis in the thermodynamic limit. Physically, this vanishing imaginary part implies that the dissipation essentially vanishes as the mode becomes entirely localized on the lossless A sublattice, and the residual imaginary part in finite systems arises strictly from finite-size boundary matching. This migration of eigenvalues onto the real axis is consistent with the previous result that the IPR scales similarly to that of extended states at large size.

## 6. Conclusions

In summary, we have derived the analytical spectrum of a Creutz ladder subject to an imaginary Stark potential. By mapping the system to a momentum-space differential equation, we identify two distinct spectral mechanisms. For energies satisfying the no-pole condition, a global phase quantization rule generates a discrete spectrum comprising an imaginary Wannier–Stark ladder and a complex connecting branch. In contrast, for real energies below the inter-cell hopping threshold ($|E|<t_{2}$), the appearance of singularities in the momentum-space wavefunction prevents the global phase winding. Instead, we show that the open boundary conditions are satisfied by a size-dependent imaginary energy component, which shifts the momentum-space singularities off the real axis to regulate the wavefunction decay at the boundaries.

Our analytical derivation predicts that this specific regularization leads to a real-space distribution characterized by size-dependent localization. We validate this description through the agreement between the theoretical pole trajectories and the numerical results. To investigate the properties of these states in the thermodynamic limit, we perform large-scale finite-size scaling analysis up to $L∼10^{9}$. These simulations provide numerical support for the “asymptotic reality” of this branch, revealing a power-law decay of the residual imaginary energy component. Furthermore, the scaling of the inverse participation ratio and fractal dimension demonstrates that these states evolve from being boundary-localized in finite systems to becoming extended in the thermodynamic limit.

This work establishes a mechanism for realizing asymptotically extended states in the presence of unbounded non-Hermitian potentials. Our findings offer a theoretical foundation for understanding the interplay between momentum-space singularities and boundary conditions in imaginary Stark systems. Experimentally, the proposed model could be realized in synthetic frequency dimensions using coupled thin-film lithium niobate resonators, extending the proposal for the Hermitian Creutz–Stark ladder [58]. In this platform, the lattice sites correspond to discrete frequency modes, and the complex hoppings are generated via electro-optic modulation. The imaginary Stark potential represents a linearly increasing, mode-dependent dissipation rate. Microscopically, this mode-dependent leakage could be engineered by coupling the B-sublattice ring resonator to an external waveguide or absorber possessing a tailored, linearly increasing transmission profile across the relevant frequency bandwidth. Future studies may extend this framework to explore the impact of many-body interactions, or generalize the theory of the critical non-Hermitian skin effect [82,83] to non-periodic systems, thereby establishing a quantitative scaling description for the imaginary energy, inverse participation ratio, and entanglement entropy of the asymptotically real branch.

## Figures and Tables

**Figure A1:**
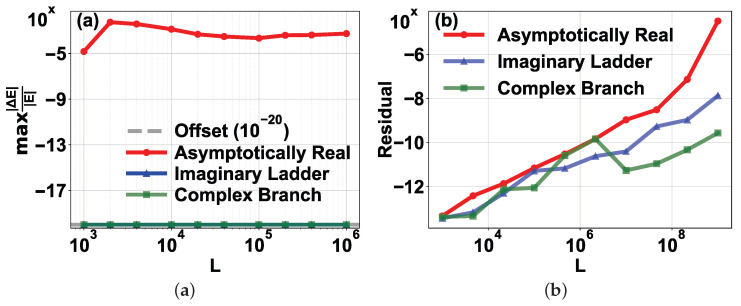
Algorithm validation and stability analysis. (**a**) Maximum relative energy error compared to standard scipy sparse solvers (calculated over 10 independent runs) for system sizes $L\approx 10^{3}$ to $10^{6}$. (**b**) Residual norm R=||(H−E)ψ||/||ψ|| as a function of system size *L* up to $10^{9}$. The three representative states used in the two subplots: the imaginary ladder (m=50), the complex branch (m=3), and the asymptotically real branch ($E\approx 0.5t_{2}$).

## Data Availability

Data are contained within the article. All figures except Figure 1 in this work were generated using the matplotlib library in Python 3.12 [84].

## Abbreviations

    The following abbreviations are used in this manuscript:

BZ	Brillouin Zone
$D_{2}$	Fractal Dimension
GBZ	Generalized Brillouin Zone
IGC	Imaginary Gap Closing
IPR	Inverse Participation Ratio
ISSE	Imaginary Stark Skin Effect
NHSE	Non-Hermitian Skin Effect
OBC	Open Boundary Condition
PBC	Periodic Boundary Condition
PT	Parity-Time

## Appendix A. Large-Scale Numerical Implementation

To accurately resolve the finite-size scaling properties up to system sizes $L\approx 10^{9}$, we extend the disk-backed Krylov subspace method developed in our previous work [58]. While the architectural framework remains similar—utilizing memory mapping (numpy.memmap [85]) to offload basis vectors to NVMe storage and a banded linear solver for the shift-invert step—the non-Hermitian nature of the current model necessitates specific adaptations.

Unlike the Hermitian case, the effective tridiagonal Hamiltonian *H* after rotation involves complex parameters and does not admit a real symmetric form. Consequently, we employ complex double-precision arithmetic (numpy.complex128) and utilize the Arnoldi algorithm [81] instead of the Lanczos algorithm for the subspace iteration. To access specific spectral regions, we solve the transformed eigenvalue problem ${\left(H-\sigma I\right)}^{-1}\psi =\nu \psi$. σ is set to be the theoretical energies for discrete energy levels (m=50 and m=3) and $0.5t_{2}$ for the asymptotically real branch. This linear inversion problem is solved using the scipy.linalg.solve_banded interface [86], which invokes the underlying LAPACK routine zgtsv optimized for complex general tridiagonal systems, which scales linearly as O(L) in both time and memory.

To ensure the reliability of this complex-arithmetic implementation, we perform benchmarks against standard sparse diagonalization libraries (scipy.sparse.linalg.eigs) for sizes where RAM allows ($L\leq 10^{6}$). Figure A1a shows the relative energy error |ΔE|/|E| for representative states. For the discrete localized states (m=3,50), the error remains near machine precision. For the continuum state ($E\approx 0.5t_{2}$), the error is slightly higher ($∼10^{-13}$) due to the extremely high density of states, which degrades the condition number of the problem.

We further monitor the numerical stability at large scales by calculating the residual R=||(H−E)ψ||/||ψ||, shown in Figure A1b. The residuals for the localized states (imaginary ladder and complex branch) remain bounded and small ($<10^{-7}$), confirming the accuracy of the eigenpairs. Conversely, the residual for the asymptotically real branch grows with *L*. As discussed in Ref. [58], this growth is physically significant: it reflects the vanishing level spacing in the continuum limit (L→∞), which causes the condition number of the shift-invert operator to diverge. Therefore, the increasing residual provides indirect evidence for the formation of a continuous spectrum in the thermodynamic limit.
